# Histone demethylase RBP2 decreases miR-21 in blast crisis of chronic myeloid leukemia

**DOI:** 10.18632/oncotarget.2859

**Published:** 2014-11-26

**Authors:** Minran Zhou, Jiping Zeng, Xiaoming Wang, Xiangyu Wang, Tao Huang, Yue Fu, Ting Sun, Jihui Jia, Chunyan Chen

**Affiliations:** ^1^ Department of Hematology, Qilu Hospital of Shandong University, Jinan, Shandong, P. R. China; ^2^ Department of Biochemistry, School of Medicine, Shandong University, Jinan, Shandong, P. R. China; ^3^ Department of Microbiology/Key Laboratory for Experimental Teratology of Chinese Ministry of Education, School of Medicine, Shandong University, Jinan, Shandong, P. R. China

**Keywords:** RBP2, miR-21, chronic myeloid leukemia, blast crisis

## Abstract

Chronic myeloid leukemia in the blastic phase (CML-BP) responds poorly to clinical treatments and is usually fatal. In this study, we found that the histone H3 lysine 4 (H3K4) demethylase RBP2 (also called JARID1A and KDM5A) is underexpressed in CML-BP. The RBP2 histone demethylase stimulates leukemia cell differentiation and inhibits cell proliferation. We identified miR-21 was directly downregulated by RBP2 and found that miR-21 downregulated PDCD4 expression in leukemia cells. By binding to miR-21 promoter and by demethylating of trimethylated H3K4 at the miR-21 locus, RBP2 downregulated miR-21 expression. This in turn activated PDCD4. In conclusion, RBP2 epigenetically downregulated miR-21 in blast transformation of CML.

## INTRODUCTION

Chronic myeloid leukemia (CML) is a myeloproliferative disorder characterized by BCR-ABL fusion gene [1]. The disease is triphasic, starting with an initial chronic phase (CP), spontaneous progression to an accelerated phase (AP) and finally a blastic phase (BP). The median survival for CML-BP patients is about 6 months. The biological mechanism responsible for promoting the transition of CML from CP to BP is poorly understood. BCR-ABL promotes the development and progression of CML CP to BP [2, 3]. However, increasing studies have suggested that factors independent of BCR-ABL, including epigenetic modifiers, participate in CML-BP progression [4-6]. However, the molecular mechanism underlying blast crisis transition remains largely unknown.

Epigenetics refers to heritable changes in gene expression that are not associated with concomitant alteration in the DNA sequence. Epigenetic modification, including DNA methylation and histone modification, is involved in the control of cell proliferation, differentiation, and apoptosis [7-9]. It is also necessary in CML progression. Transcription factor AP-2 alpha (TFAP2A) and Early B-Cell Factor 2 (EBF2) were identified as epigenetic molecular markers in Acute lymphoblastic leukemia (ALL) and CML-BP. The markers showed increased methylation in CML-BP as compared with CP [10]. Furthermore, preferentially expressed antigen of melanoma (PRAME), a type of Tumor associated antigens (TAA) gene, is overexpressed in CML-BP because of hypomethylation. This kind of regulation is critical for the transformation from CP to BP [11]. Moreover, some reports indicated that histone deacetylase inhibitors could induce cell cycle arrest and cell apoptosis in CML-BP cells [12-14].

Histone methylation is a type of histone modification that epigenetically controls gene expression at the genome-wide level [15] via histone-modifying enzymes, including methyltransferases and demethylases. The tumor suppressor Retinoblastoma-interacting zinc-finger protein 1 (RIZ1), a PR domain methyltransferase, is downregulated in CML-BP [16]. Whether histone demethylases are crucial for CML progression has not been fully elucidated. Retinoblastoma binding protein 2 (RBP2), a member of the JARID family proteins, has histone demethylase activity and specifically demethylates tri- and dimethylated lysine 4 of histone 3 (H3K4) [17-19].

MicroRNAs (miRNAs) are small non-coding RNAs (snRNAs) that downregulate gene expression by directly binding to the 3′ untranslated regions (3′UTR) of target gene mRNA, which results in translational inhibition or degradation [20]. Dysregulation of miRNAs is involved in many kinds of human cancers, including leukemia [20, 21] and is related to cell development, proliferation, differentiation, and apoptosis [22]. Recently, in examining epigenetic regulation of miRNAs in tumorigenesis [23-25], miR-17-92 was found epigenetically downregulated by JARID1B. Its overexpression resulted in abnormal proliferation, blockade of differentiation and decreased apoptosis, which led to acute myeloid leukemia [25].

MiR-21 was found to be an oncogenic miRNA in human cancers [26, 27]. Anti-miR-21 oligonucleotide could sensitize leukemic K562 cells to arsenic trioxide treatment by inducing apoptosis [28, 29]. However, whether epigenetic regulation of miR-21 is involved in CML progression is unknown.

We aimed to define the molecular mechanism of histone demethylase RBP2 directly and epigenetically downregulating miR-21. In CML, low RBP2 expression could not repress miR-21 expression, which promoted the transition of CML from CP to BP.

## RESULTS

### RBP2 induces leukemia cell differentiation

**Figure 1:**
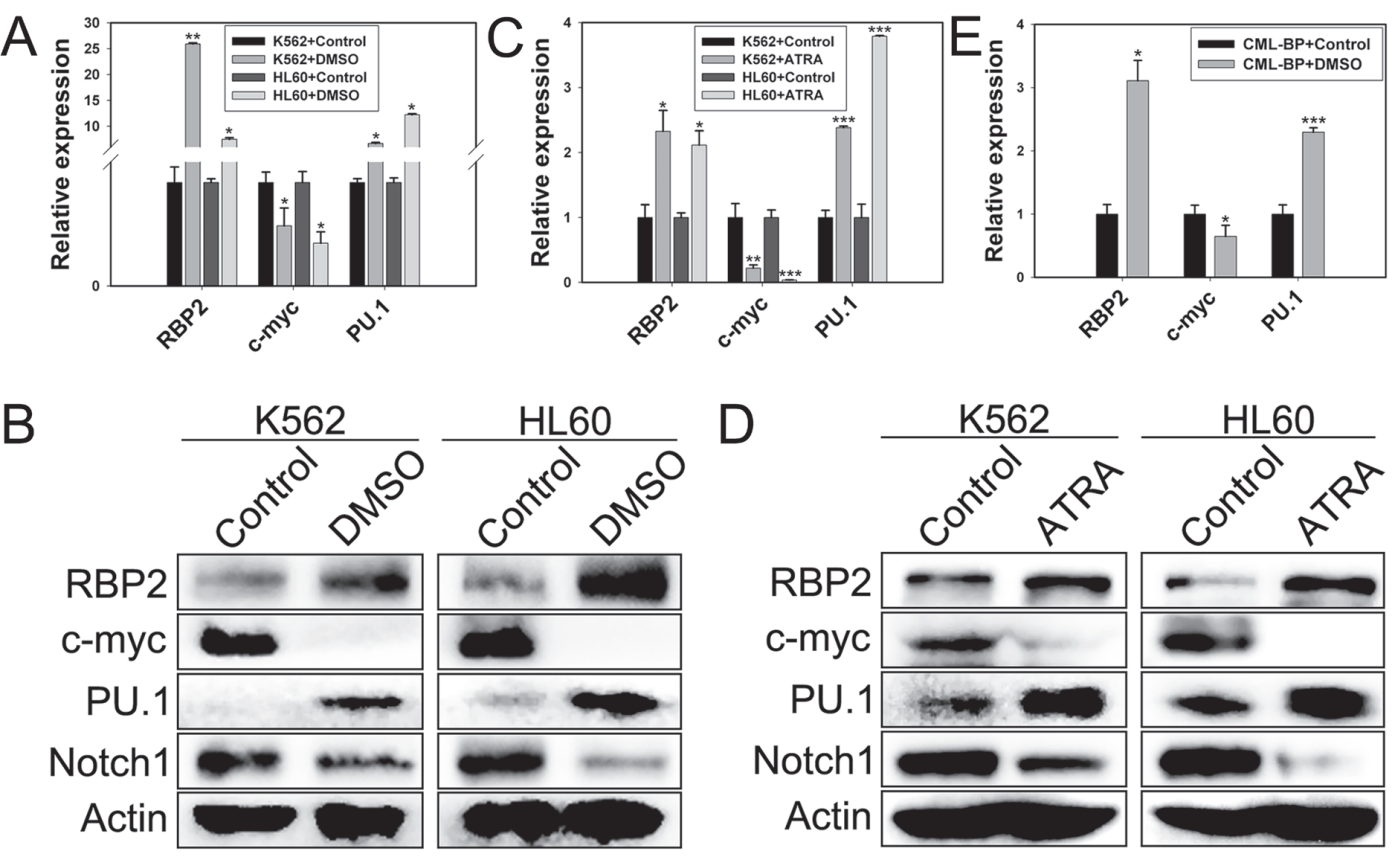
RBP2 induces cell differentiation (A) The mRNA expression of RBP2 in differentiated K562 and HL60 leukemia cells induced by DMSO. Quantitative RT-PCR (qRT-PCR) analysis of the mRNA levels of RBP2, c-myc, and PU.1 and loading control β-actin. Data are mean ± SEM of 3 independent experiments. (B) Western blot analysis of RBP2, c-myc, PU.1 and Notch1 protein level after treatment with DMSO. β-actin was a loading control. (C) qRT-PCR analysis of RBP2, c-myc, and PU.1 mRNA expression and the loading control β-actin in differentiated cells induced by ATRA. Data are mean ± SEM of 3 independent experiments. (D) Western blot analysis of RBP2, c-myc, PU.1 and Notch1 protein level after treatment with ATRA. β-actin was a loading control. (E) The mRNA expression of RBP2, c-myc, and PU.1 in differentiated primary cells induced by DMSO. The results are from 3 independent experiments. *P< 0.05, **P< 0.01, ***P< 0.001.

We determined the requirement for RBP2 in differentiation of leukemia cells by inducing granulocytic differentiation *in vitro*. RBP2 mRNA and protein levels were increased (Figure 1A-D) in K562 and HL60 cells undergoing granulocytic differentiation induced by DMSO or ATRA (Supplement Figure 1). As expected, the expression of differentiation-related c-myc and Notch1 was downregulated and PU.1 expression was upregulated after terminal differentiation (Figure 1A-D). In addition, CML-BP primary cells were treated with DMSO for 7 days, with similar results (Figure 1E). Therefore, RBP2 induces leukemia cell differentiation.

### Ectopic expression of RBP2 inhibits leukemia cell proliferation

**Figure 2:**
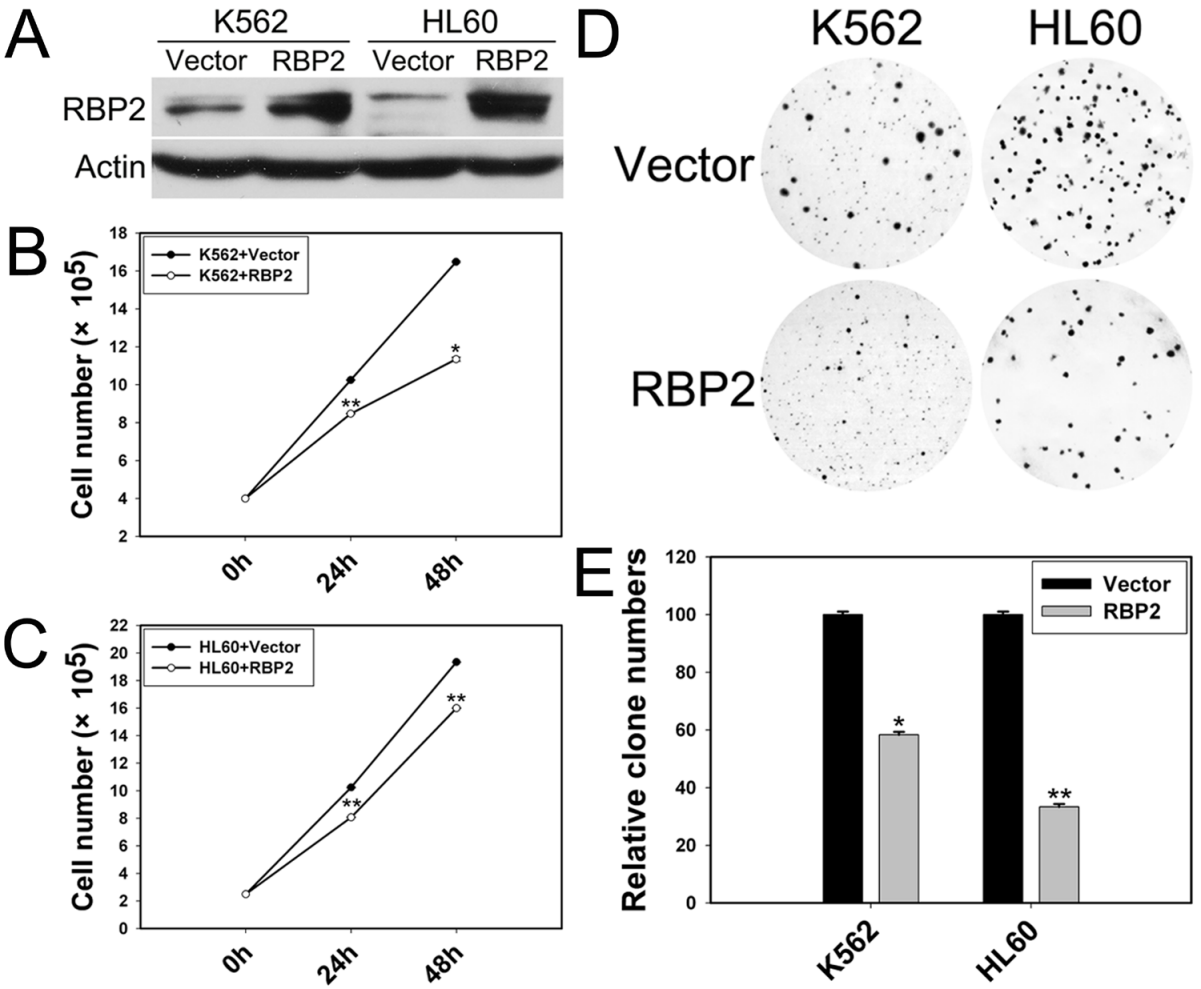
RBP2 inhibits the cell proliferation (A) Western blot analysis of protein level of RBP2 with RBP2 expression plasmid. β-actin was a loading control. (B, C) Proliferation of K562 and HL60 cells after transfection with RBP2 expression plasmid. (D, E) Foci formation of K562 and HL60 cells after transfection with RBP2 expression plasmid. The results are from 3 independent experiments. *P< 0.05, **P< 0.01.

We explored the potential role of RBP2 in leukemia cell proliferation by transfecting RBP2 expression plasmid into K562 and HL60 cells. RBP2 protein level was increased significantly in K562 and HL60 cells (Figure 2A). As well, K562 and HL60 cells transfected with RBP2 expression plasmid proliferated at a slower rate than with vector transfection (Figure 2B, C), and their colony-formation ability was impaired (Figure 2D, E). Thus, RBP2 inhibited proliferation of leukemia cells.

### MiR-21 is downregulated by RBP2 in leukemia cells and CML primary cells

**Figure 3:**
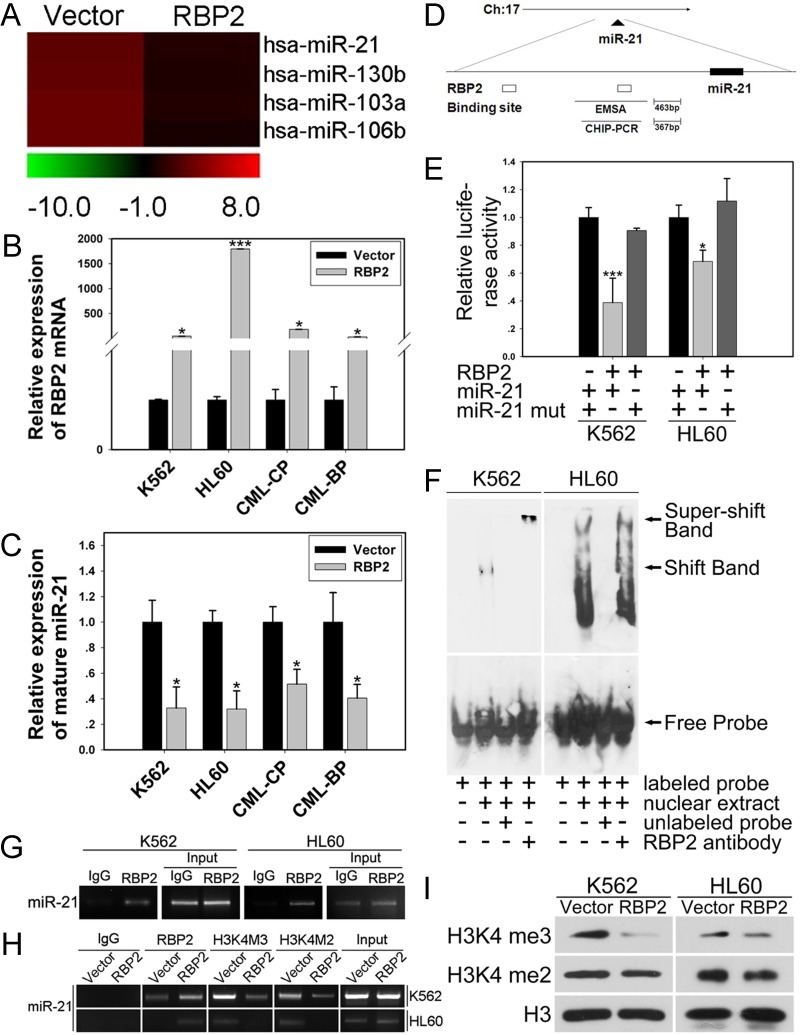
MiR-21 is directly and epigenetically downregulated by RBP2 (A) miRNA expression analysis of the level of miR-21 in K562 cells with RBP2 expression plasmid. (B) qRT-PCR analysis of RBP2 mRNA level after transfection with RBP2 expression plasmid in K562, HL60 and CML-CP and -BP primary cells for 48 h. The primary cells were from the bone marrow of CML-CP and -BP patients. Data are mean ± SEM of 3 independent experiments. (C) qRT-PCR analysis of miR-21 and the loading control U6 snRNA after transfection with RBP2 expression plasmid. Data are mean ± SEM of 3 independent experiments. (D) Predicted binding site of RBP2 in the miR-21 promoter. (E) MiR-21 promoter and miR-21 mutated activity with RBP2 expression plasmid transfection in K562 and HL60 cells. Luciferase activities were determined at 48 h and normalized by Renilla luciferase activity. (F) EMSA assay for the binding of RBP2 to miR-21 promoter. (G) ChIP assay for binding RBP2 to miR-21 promoter in K562 and HL60 cells. (H) The binding of RBP2 and H3K4me3/2 to miR-21 promoter after RBP2 expression plasmid transfection. (I) Western blot analysis of global H3K4me3/2 protein expression in K562 and HL60 cells after transfection with RBP2 expression plasmid. The results are from 3 independent experiments. *P< 0.05, ***P < 0.001.

Increasing studies have shown that miRNAs are pivotal in leukemogenesis. To investigate the mechanisms by which RBP2 promotes cell differentiation and inhibits proliferation, we examined the effect of RBP2 overexpression on miRNA expression profiles. We compared miRNA expression levels in RBP2 and control plasmid-treated K562 cells by miRNA microarray analysis and found miRNA genes that were up- or downregulated by RBP2 overexpression. miR-21 was significantly downregulated by RBP2 overexpression (Figure 3A). miR-21 expression was significantly reduced with upregulated RBP2 in K562 and HL60 cells (Figures 3B, C) and in primary cells from the bone marrow of CML-CP and CML-BP patients (Figures 3B, C). Furthermore, with RBP2 overexpression, the level of RBP2 was lower in CML-BP than CML-CP cells (Figure 3B), which suggests that RBP2 expression was decreased during CML progression. Therefore, miR-21 is downregulated by RBP2, so it may be a target gene of RBP2.

### RBP2 directly targets the promoter of miR-21 to repress its expression depending on histone demethylase activity

To determine whether miR-21 is a direct target of RBP2, we identified a potential RBP2 binding site in the promoter of miR-21 (Figure 3D). miR-21 promoter activity was significantly decreased after RBP2 overexpression in K562 and HL60 cells, with no change in miR-21 promoter activity on mutation of the binding site (Figure 3E).

We determined the association of RBP2 with miR-21 promoter. In K562 and HL60 cells, DNA-protein complexes were present in nuclear extracts (Figure 3F, the shift band in lane 2 in each cell line). To further confirm whether the shift band was specific to the RBP2 complex, we performed a competition assay. The shift band could be abolished by 100-fold excess unlabeled miR-21-RBP2 probe (Figure 3F). The decrease in shift band intensity and increase in super-shift band intensity indicated that RBP2 binds to miR-21-RBP2 probe *in vitro*.

To determine whether RBP2 actually binds to human miR-21 promoter in intact cells, we performed chromatin immunoprecipitation assay. In K562 and HL60 cells, RBP2 bound to the promoter region of the miR-21 promoter (Figure 3G). Furthermore, in cells treated with RBP2 expression plasmid, RBP2 overexpression increased its association with miR-21 promoter sequences (Figure 3H). RBP2 overexpression also remarkably reduced H3K4 trimethylation and moderately reduced H3K4 dimethylation at the proximal promoter region of miR-21 (Figure 3H). Therefore, RBP2 causes loss of H3K4me3 and reduced H3K4me2 by binding to the miR-21 promoter region in K562 and HL60 cells.

### Global decrease in tri- and dimethylated H3K4 in RBP2-overexpressed K562 and HL60 cells

Because RBP2 is a histone demethylase specifically targeting H3K4 tri- and dimethylation, we examined the change in global levels of tri- and dimethylated H3K4 in K562 and HL60 cells transfected with RBP2 expression plasmid and found a substantial decrease in H3K4 trimethylation and moderate decrease in H3K4 dimethylation in these RBP2-overexpressed cells (Figure 3I).

### RBP2 induces cell differentiation and inhibits cell proliferation depending on miR-21

**Figure 4:**
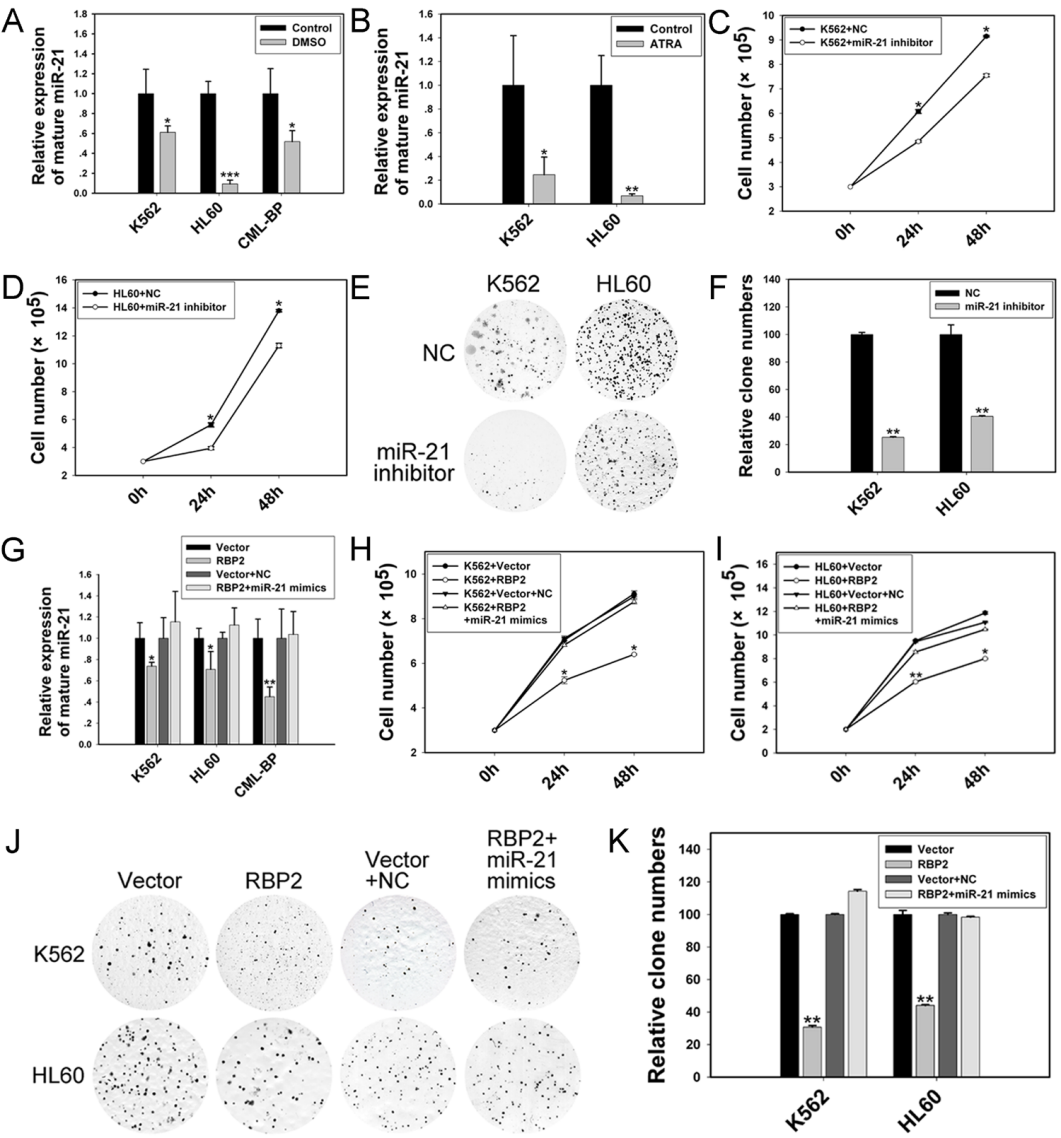
RBP2-mediated differentiation and proliferation depends in part on miR-21 in K562 and HL60 cells (A, B) qRT-PCR analysis of level of miR-21 in differentiated cells induced by DMSO or ATRA. Data are mean ± SEM of 3 independent experiments. (C, D) Cell proliferation of K562 and HL60 cells after transfection with miR-21 inhibitor. (E, F) Foci formation of K562 and HL60 cells after transfection with miR-21 inhibitor. (G) qRT-PCR analysis of miR-21 and the loading control U6 snRNA 48 h after RBP2 expression plasmid transfection without or with miR-21 mimics. Data are mean ± SEM 3 of independent experiments. (H, I) Cell proliferation assay of K562 and HL60 cells after RBP2 expression plasmid transfection without or with miR-21 mimics for 24 and 48 h. (J, K) Colony-formation assay of K562 and HL60 cells after RBP2 expression plasmid transfection without or with miR-21 mimics for 48 h. The results are from 3 independent experiments. *P< 0.05, **P< 0.01, ***P < 0.001.

**Figure 5:**
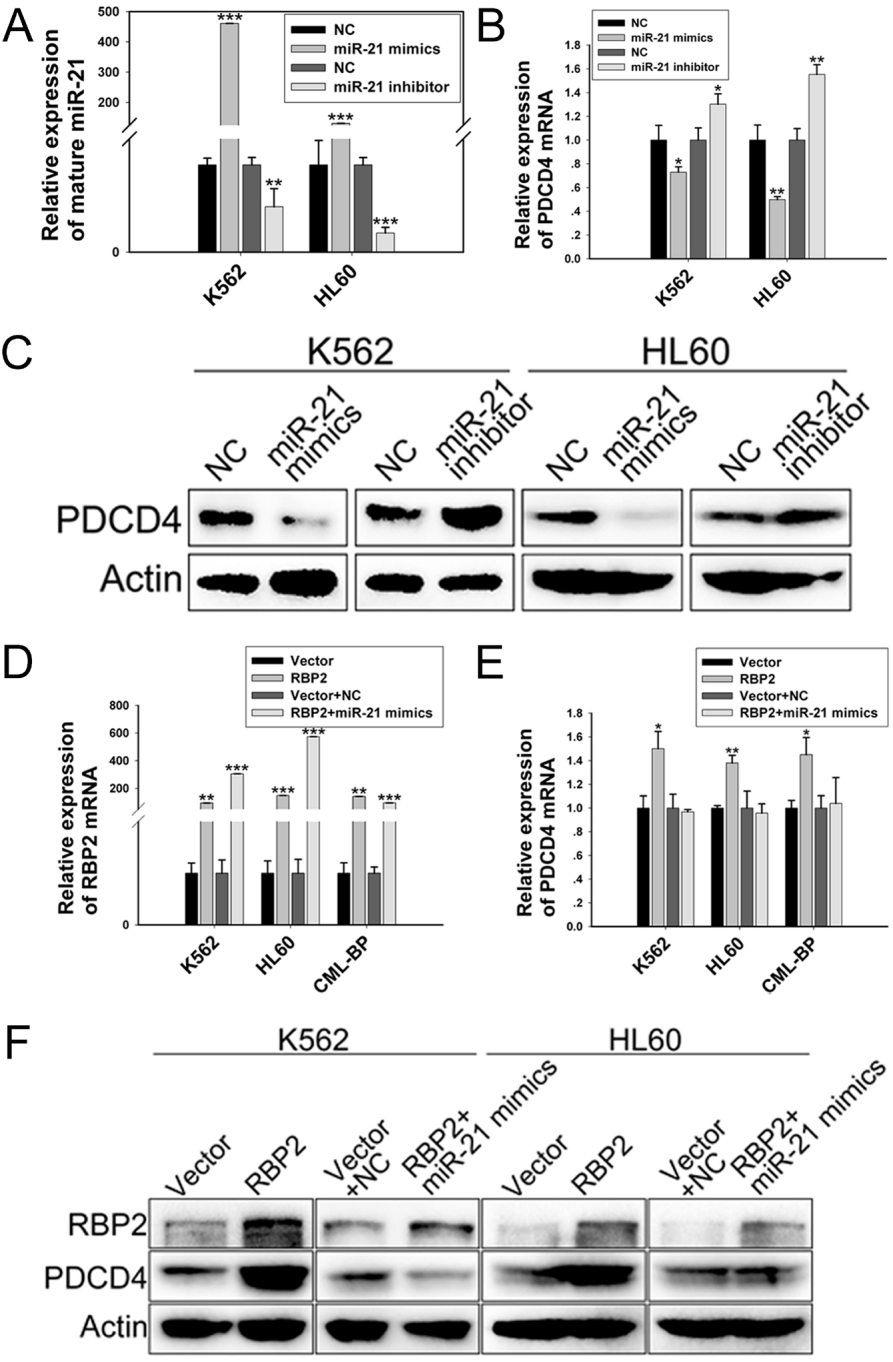
PDCD4 is directly downregulated by miR-21 qRT-PCR analysis of the level of (A) mature miR-21 and (B) PDCD4 after transfection with miR-21 mimics or inhibitor. Data are mean ± SEM of 3 independent experiments. (C) Western blot analysis of PDCD4 protein level with miR-21 mimics or inhibitor treatment. β-actin was a loading control. qRT-PCR analysis of the mRNA level of (D) RBP2 and (E) PDCD4 with RBP2 expression plasmid transfection without or with miR-21 mimics. Data are mean ± SEM of 3 independent experiments. (F) Western blot analysis of RBP2 and PDCD4 protein level with RBP2 expression plasmid transfection without or with miR-21 mimics. β-actin was a loading control. The results are from 3 independent experiments. *P< 0.05, **P< 0.01, ***P < 0.001.

Given that ectopic expression of RBP2 triggered inhibition of miR-21 expression in K562 and HL60 cells, we hypothesized a link between reduced miR-21 expression and defective clonogenesis of RBP2-expressed cells. We explored the effect of miR-21 on cell differentiation and proliferation. When K562 and HL60 cells were induced to undergo granulocytic differentiation by DMSO or ATRA (Supplemental Figure 1), the level of miR-21 was decreased (Figure 4A, B), which suggests that miR-21 blocks cell differentiation. In addition, miR-21 inhibitor significantly decreased the level of miR-21 in K562 and HL60 cells (Figure 5A), accompanied by reduced rate of cell proliferation (Figure 4C, D) and impaired colony-formation ability (Figure 4E, F), so miR-21 stimulates cell proliferation.

K562 and HL60 cells were treated with both RBP2 expression plasmid and miR-21 mimics, then foci formation was analyzed. The mRNA and protein levels of RBP2 were elevated in K562 and HL60 cells transfected with RBP2 expression plasmid without and with miR-21 mimics (Figure 5D, F). The RBP2 overexpression-reduced miR-21 level was efficiently rescued by miR-21 mimics transfection (Figure 4G). miR-21 overexpression significantly abrogated the inhibited proliferation mediated by RBP2 overexpression (Figure 4H-K). Therefore, induction of differentiation and suppression of proliferation by RBP2-overexpressing K562 and HL60 cells depends at least in part on the repression of miR-21 levels by RBP2.

### Programmed cell death 4 (PDCD4), which promotes cell differentiation and inhibits cell proliferation, is directly downregulated by miR-21

To investigate the mechanism by which miR-21 blocks cell differentiation and stimulates proliferation, we identified PDCD4 as a target of miR-21. After transfection with miR-21 mimics and inhibitor, the expression of miR-21 was upregulated and downregulated, respectively (Figure 5A). The mRNA and protein levels of PDCD4 were decreased with miR-21 mimics and increased with miR-21 inhibitor treatment (Figure 5B, C). Therefore miR-21 decreased PDCD4 expression in K562 and HL60 cells. Transfection with RBP2 expression plasmid increased mRNA and protein levels of PDCD4, and miR-21 mimic treatment reversed the RBP2-overexpression-induced PDCD4 (Figure 5E, F), which suggests that RBP2 increased PDCD4 expression via miR-21.

**Figure 6:**
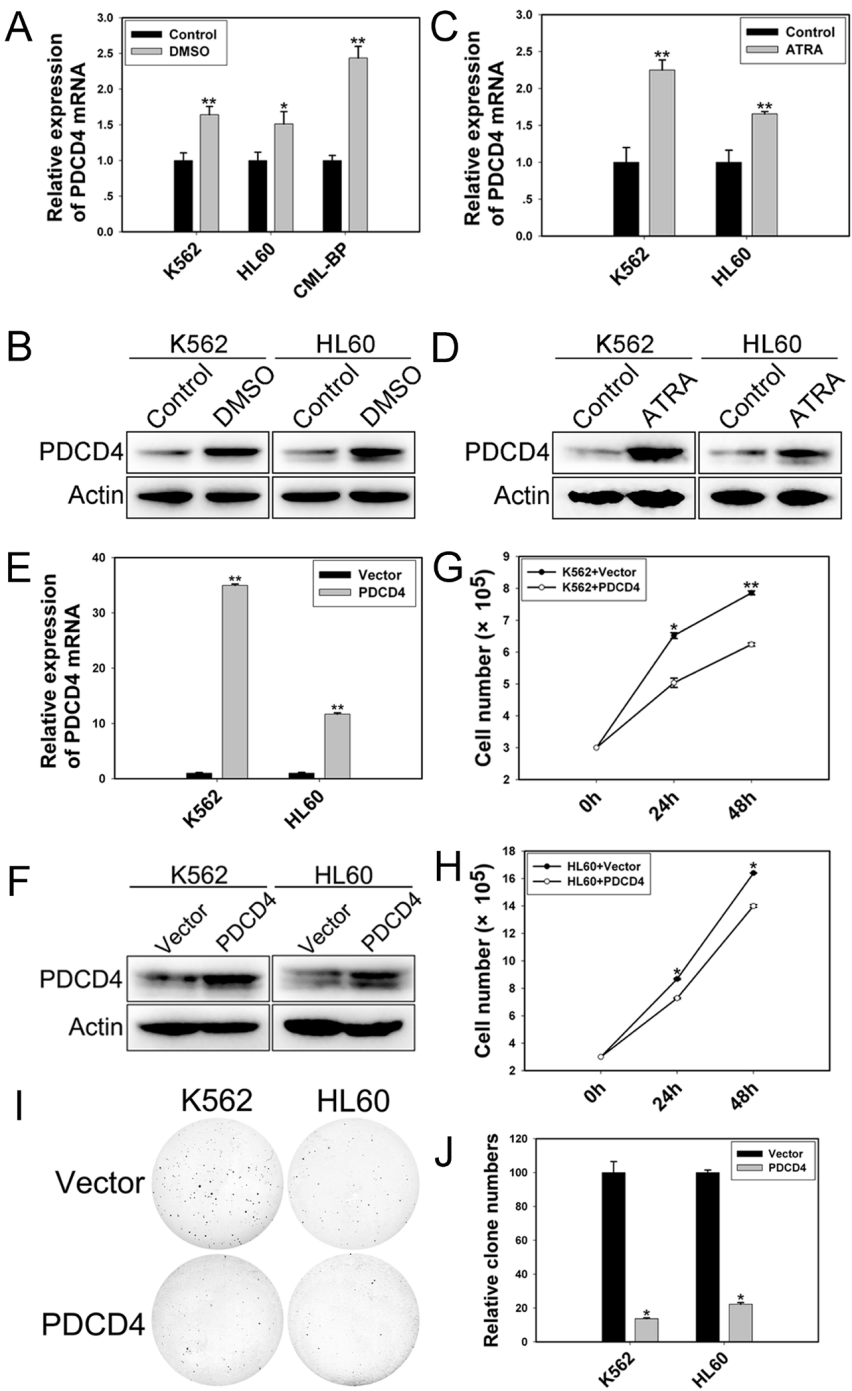
PDCD4 promotes cell differentiation and inhibits proliferation qRT-PCR and western blot analysis of the mRNA and protein levels of PDCD4 in (A, B) differentiated cells induced by DMSO and (C, D) induced by ATRA. (E) qRT-PCR analysis of the mRNA expression of PDCD4 with PDCD4 expression plasmid transfection. Data are mean ± SEM of 3 independent experiments. (F) Western blot analysis of the protein level of PDCD4 with PDCD4 expression plasmid transfection. β-actin was a loading control. (G, H) Cell proliferation assay of K562 and HL60 cells with PDCD4 expression plasmid transfection after 24 and 48 h. (I, J) Colony-formation assay of K562 and HL60 cells with PDCD4 expression plasmid transfection at 48 h. The results are from 3 independent experiments. *P< 0.05, **P< 0.01.

K562 and HL60 cells induced to undergo granulocytic differentiation by DMSO or ATRA (Supplemental Figure 1) showed upregulated PDCD4 level (Figure 6A-D). Moreover, with ectopic expression of PDCD4 (Figure 6E, F), K562 and HL60 cells proliferated at a slow rate (Figure 6G, H) and showed impaired colony-formation ability (Figure 6I, J).

### Low RBP2 levels and high miR-21 levels in CML-BP

**Figure 7:**
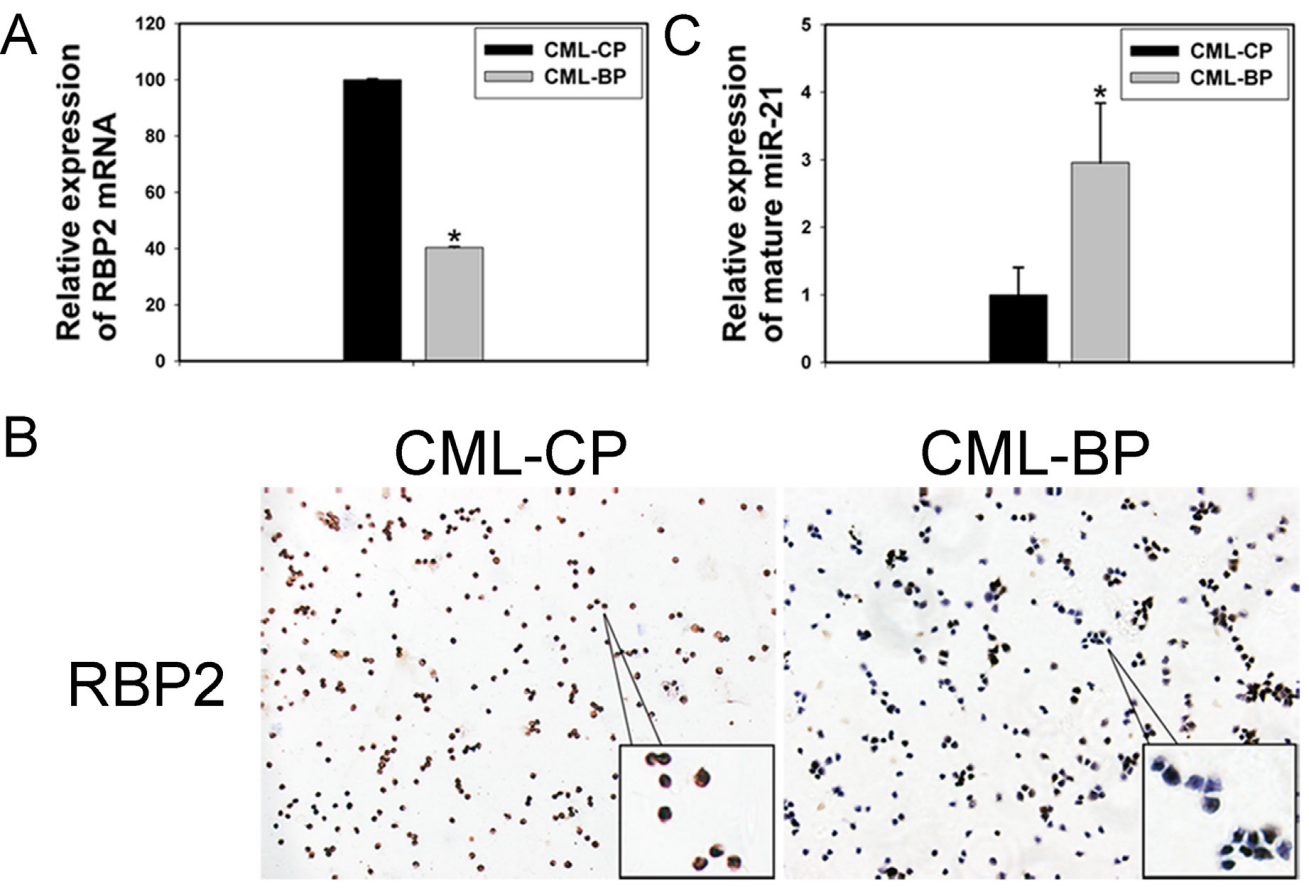
The expression of RBP2 and miR-21 in 26 patients with chronic myeloid leukemia in the chronic phase (CML-CP) and in 18 with blastic phase (CML-BP) (A, B) qRT-PCR and IHC analysis of RBP2 mRNA and protein levels. Data are mean ± SEM. (C) qRT-PCR analysis of the relative expression of mature miR-21 in CML-CP patients and CML-BP patients. Human CML-CP and -BP cells were from bone marrow of patients. Data are mean ± SEM. The results were confirmed by 3 independent experiments. *P< 0.05.

To investigate whether RBP2 was critical in CML progression, we measured RBP2 mRNA and protein levels in bone-marrow samples from patients with newly diagnosed CML-CP or CML-BP. The clinical characteristics of CML patients are in Table 1. RBP2 mRNA and protein levels were lower in CML-BP than CML-CP samples (Figure 7A, B). Therefore, RBP2 is underexpressed during CML progression. miR-21 level was higher in CML-BP than CML-CP samples (Figure 7C).

**Table 1:** Patient characteristics

Characteristic		CML-CP patients (n=26)	CML-BP patients (n=18)
Gender	Male	16	13
	Female	10	5
Age(years)	Median	46	45
	Range	21-80	28-69
WBC,×10^9^/L	Median	212.6	71.1
	Range	2.31-505	2.7-300
Hemoglobin, g/L	Median	97.8	80.6
	Range	68-140	45-124
Platelet count, ×10^9^/L	Median	297	185
	Range	32-547	1-840

## DISCUSSION

Recent studies suggest that epigenetic regulation is involved in the progression of CML from CP to BP [10-14, 16]. However, the modification of histone demethylase in this kind of disease progression has yet to be described. In this study, we showed that the histone demethylase RBP2 is downregulated in CML-BP as compared with CML-CP. Furthermore, RBP2 induced cell differentiation and inhibited cell proliferation. Therefore, the underexpression of RBP2 may promote the pathogenesis of CML-BP.

Several studies have shown that miR-21 is an oncogenic miRNA in various cancers such as lung cancer [30, 31], gastric cancer [31], lymphoma [32], and chronic lymphocytic leukemia [33]. miR-21 blocks cell differentiation [34-37] and promotes cell proliferation [38]. We found that miR-21 plays an oncogenic role in CML progression by blocking cell differentiation and by excessive proliferation. Moreover, miR-21 overexpression could recover the RBP2-inhibited proliferation. Therefore, we discovered a novel relationship between a histone demethylase and another epigenetic modifier, miRNA.

The AT-rich interaction domain of RBP2 can recognize a specific DNA sequence CCGCCC [39] contained in the promoter region of miR-21. We found that RBP2 binds to the proximal promoter of miR-21 and downregulates its transcription. RBP2 overexpression significantly downregulated miR-21 expression in K562, HL60 and CML primary cells and inhibited miR-21 promoter activity. However, when the binding site was mutated, the inhibition disappeared. Furthermore, ChIP assay showed that RBP2 directly bound to the promoter sequence of miR-21.

As a histone demethylase specific for di- and trimethylated H3K4, RBP2 upregulates the expression of cyclins D1, E1 and integrin-β1 (ITGB1) etc. or suppressing the expression of cyclin-dependent kinase inhibitor (CDKIs) p21(CIP1), p27(kip1) etc. by altering H3K4 methylation at the promoters of target genes [40, 41]. Here, we provide evidence that RBP2 decreased the expression of miR-21 epigenetically. RBP2 overexpression increased its occupancy on the miR-21 promoter, significantly decreased H3K4 trimethylation and moderately decreased H3K4 dimethylation locally. Therefore, RBP2 can directly and epigenetically downregulate miR-21.

In addition, we illustrate that low RBP2 expression promoted clinical CML progression. However, the reason for RBP2 downregulation in CML is still unknown. Further studies are needed to uncover these intrinsic mechanisms.

Genes related to differentiation and proliferation are often aberrantly expressed in CML progression. PDCD4 is a tumor suppressor protein that inhibits cell proliferation [42], induces apoptosis [43], suppresses autophage [44], and promotes differentiation [35, 45, 46]. In K562 and HL60 cells, PDCD4 expression was downregulated or upregulated with miR-21 mimics or inhibitor treatment, respectively. In differentiated cells, PDCD4 expression was elevated. Therefore, PDCD4 mediates the effect of miR-21 on differentiation, but whether it is the only gene mediating this effect remains for further research.

**Figure 8:**
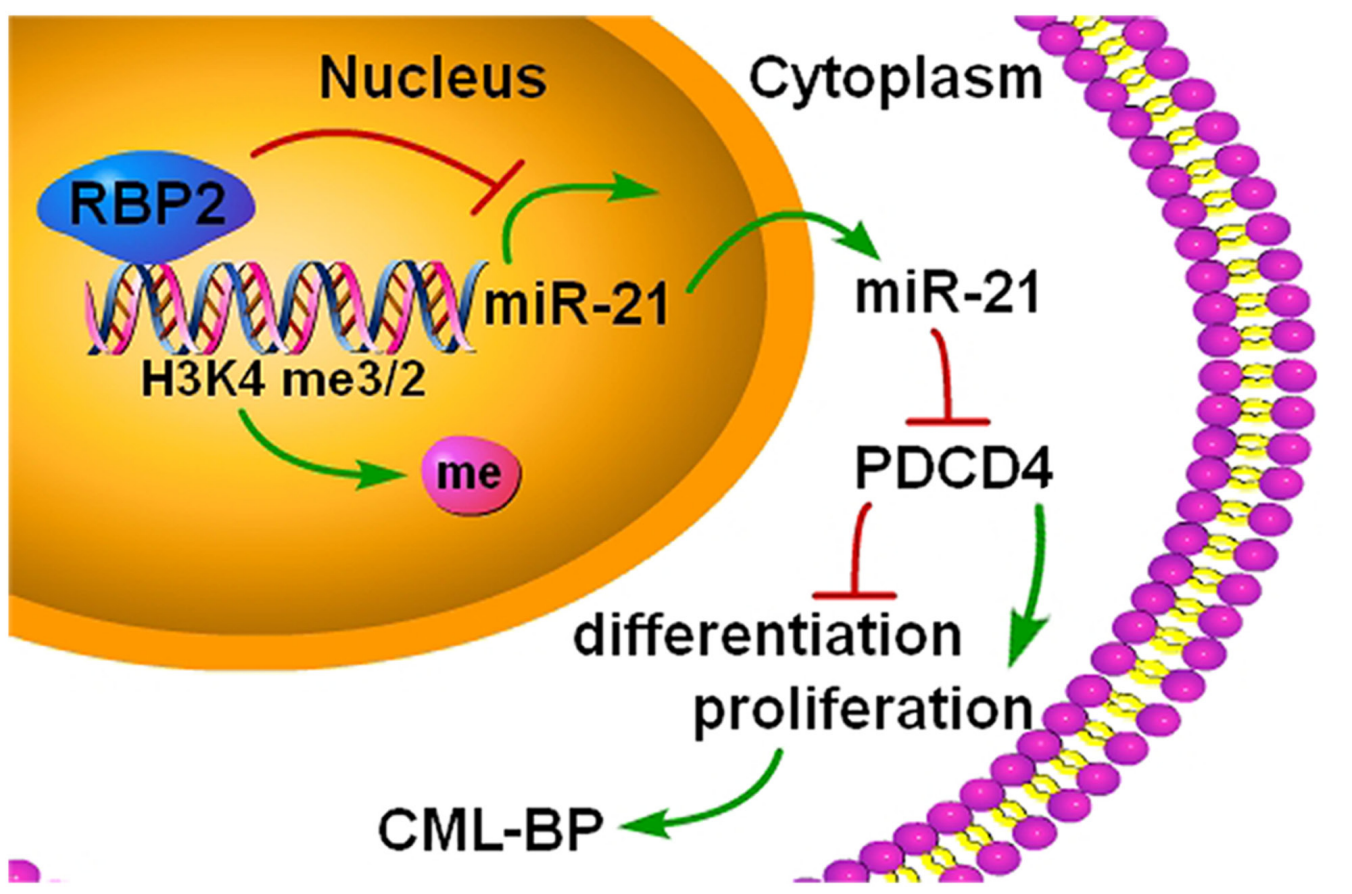
A summary of the findings A new epigenetic mechanism involved in the pathogenesis of CML-BP: by binding to promoter and demethylation of trimethylated H3K4 at the miR-21 locus, RBP2 downregulates miR-21 expression, which in turn activates PDCD4. In CML progression, low levels of RBP2 cannot repress the expression of miR-21, which decreases PDCD4 expression to block cell differentiation and stimulate cell proliferation.

Overall, we found a new epigenetic mechanism involved in the pathogenesis of CML-BP (Figure 8). Because CML-BP responds poorly to treatment and is usually fatal, we need to uncover mechanisms and find new drug targets. Here we found that the histone demethylase RBP2 is underexpressed in CML-BP. Low RBP2 expression could not repress miR-21 expression, which promoted the transition of CML from CP to BP. RBP2 could be a useful biological marker in CML progression.

## METHODS

### Human bone-marrow samples

Patient bone-marrow samples were from patients with newly diagnosed CML-CP (n=26) and CML-BP (n=18) from the Department of Hematology, Qilu Hospital of Shandong University, Jinan, China. Mononuclear cells were isolated from samples by Ficoll-Hypaque density-gradient centrifugation, then stored at −80°C. The study was approved by the Ethics Committee of Shandong University School of Medicine.

### Cell culture and transfection

K562 and HL60 cells were maintained in our laboratory. Cells were cultured at 37 °C, 95% air and 5% CO_2_ in RPMI 1640 containing 10% heat-inactivated fetal bovine serum without antibiotics (FBS; Gibco, Carlsbad, CA, USA) on 6/24-well plates for 18 to 24 h. Cells were transfected with RBP2 expression plasmid and/or miR-21 mimics (miR10000076-1-2) and inhibitor (miR20000076-1-2; Ribobio, Guangzhou, China) by use of Lipofectamine 2000 (Invitrogen, Carlsbad, CA, USA). Cells (1×10^5^/ml) were induced to differentiate by the addition of 2.5% or 1.25% dimethy sulfoxide (DMSO) or 1 μM ATRA (Sigma-Aldrich).

### RNA extraction and quantitative real-time PCR

Total RNA from human bone-marrow samples and cells was extracted by using Trizol (Invitrogen, Carlsbad, CA, USA). Complementary DNA (cDNA) was synthesized with random primers and MMLV reverse transcriptase (Fermentas, Canada). Level of RBP2 mRNA was normalized to that of human β-actin. qRT-PCR involved the TaqMan miRNA assay kit (Applied Biosystems, Foster City, CA, USA) with U6 snRNA used as a control. The level of miR-21 was normalized to that of U6 snRNA. The probes for RBP2 and miR-21 (Applied Biosystems) were Hs00231908_m1 and 000397, respectively. The mRNA level of PDCD4, c-myc and PU.1 was determined by RT and SYBR-Green real-time PCR assay (TaKaRa, Japan). qRT-PCR involved ABI7500 sequence detection (Applied Biosystems, Foster City, CA, USA). The PCR primers are in Table 2. Gene expression was normalized to that of β-actin. Expression was calculated by the 2^−ΔΔCt^ method.

**Table 2:** PCR primers

PDCD4	5′- CAGTTGGTGGGCCAGTTTATTG-3′ (Forward) 5′- AGAAGCACGGTAGCCTTATCCA-3′ (Reverse)
PU.1	5′-AGTCATGCCTGTAGCTCCATTCT-3′ (Forward) 5′-TCTCTCTACATTGCCTGGGTTTC-3′ (Reverse)
c-myc	5′-TACCCTCTCAACGACAGCAGCTCGCCCAACTCCT-3′ (Forward) 5′-TCTTGACATTCTCCTCGGTGTCCGAGGACCT-3′ (Reverse)
β-actin	5′-AGTTGCGTTACACCCTTTCTTG-3′ (Forward) 5′-CACCTTCACCGTTCCAGTTTT-3′ (Reverse)

### Western blot analysis

Cells were lysed in RIPA lysis buffer with proteinase inhibitor (Biocolor BioScience & Technology, Shanghai). Total cellular proteins were separated by SDS-PAGE and transferred to PVDF membranes, which were probed with antibodies against RBP2 (1:1000, Abcam), PDCD4 (1:1000, Cell Signalling), PU.1 (1:200, Abcam), c-myc (1:500, Santa Cruz Biotechnology), Notch1 (1:1000, Epitomics) and β-actin (1:10000, Sigma) overnight at 4°C followed by horseradish peroxidase-labeled goat-anti-rabbit IgG (1:6000, Abcam) for 1 h. The signals were detected by enhanced chemiluminescence. β-actin was a loading control. For western blot analysis of histone H3K4 di- and trimethylation, a total histone fraction was isolated from nuclei by dilute acid extraction. The membranes were probed with the antibodies against di- and trimethylated H3K4 (1:3000, Abcam). H3 was a loading control (1:10000, Abcam).

### Flow cytometry

K562 and HL60 cells were seeded in 6-well plates for treatment with DMSO or ATRA for various times. Then 10^6^ cells were harvested, washed twice with PBS and resuspended in 100 μl phosphate buffered saline (PBS) with 20 μl antibodies against CD11b and CD13 (BD Pharmingen) for 30 min in the dark, then washed with PBS, resuspended in PBS and analyzed by flow cytometry on a FACScan (Becton Dickinson).

### Immunostaining

Mononuclear cells isolated from patient bone-marrow samples were used to prepare cytospins with glass slides treated by Poly-L-lysine (PLL), then fixed in ice-cold acetone. Samples were stained with anti-RBP2 antibody (1:150, Abcam) overnight at 4°C, then horseradish peroxidase-conjugated secondary antibody for 30 min.

### Cell proliferation assay

Cells were seeded in 6-well plates and transfected with RBP2 expression plasmid without or with miR-21 mimics or inhibitor. Relative cell numbers were determined at 24 and 48 h by counting surviving cells.

### Soft agar assay of colony-formation

Cells were treated as in the cell proliferation assay. In total, 1 ml of 1% agar in complete DMEM was plated as the basal layer in 6-well plates. Cells (1.5×10^3^) in complete medium containing 0.4% or 0.3% agar were seeded on the basal layer. Plates were incubated at 37°C in a CO_2_ incubator for 14 days. Opaque and dense colonies were examined and counted on day 14 day.

### Luciferase reporter assay

The miR-21 reporter construct harboring its promoter sequences was previously described [47]. To test the effect of RBP2 on promoter activity, we transfected K562 and HL60 cells with RBP2 expression plasmid on day 1 and wild-type/mutant miR-21 promoter reporter plasmid on the following day. Thymidine kinase promoter was cotransfected to monitor transfection efficiency. After 48 h, luciferase activity was determined by the Dual-Luciferase Reporter Assay System (Promega). Luciferase activity of the miR-21 promoter reporter was normalized to thymidine kinase renilla activity.

### Electrophoretic mobility shift assays (EMSA)

Nuclear proteins from K562 and HL60 cells were prepared. The reaction mixtures containing nuclear extracts were incubated with digoxigenin-labeled probes and/or RBP2 antibody in reaction buffer for 25 min at room temperature. Samples underwent electrophoresis in 5% nondenaturing polyacrylamide gel and were transferred to nylon membrane (Millpore). For competition analysis, 100-fold excess of unlabeled probes was included. Digoxigenin-labeled probes were 5′-attgagaaagaccgcccccgcccccgccctct-3′ (forward); 5′-agagggcgggggcgggggcggtctttctcaat-3′ (reverse).

### Chromatin immunoprecipitation (ChIP)

ChIP assay involved the Millipore ChIP assay protocol. K562 and HL60 cells untreated or transfected with RBP2 expression or control plasmid were cross-linked by incubation in 37% formaldehyde solution for 10 min at 37°C and sonicated to develop soluble chromatin with DNA fragments from 200 to 1000 bp. DNA was purified from chromatin fragment immunoprecipitated with antibodies against RBP2 (Abcam) and di- and trimethylated H3K4 (Abcam), and used for PCR amplification. The PCR primers for miR-21 promoter were 5′-TCCCAATCATCTCAGAACAAG-3′ (forward) and 5′-AAGTCCCACATTTATCACCAC-3′ (reverse).

### Statistical analysis

Data are expressed as mean ± SEM from 3 independent experiments. Differences were calculated by two-tailed Student's *t* test or one-way ANOVA by use of SPSS 13.0 (SPSS Inc., Chicago, IL). *P*< 0.05 was considered statistically significant.

## SUPPLEMENTARY MATERIAL AND FIGURE


