# Supplementary material for: Histone demethylase RBP2 decreases miR-21 in blast crisis of chronic myeloid leukemia
Source: Oncotarget. 2014 Nov 26;6(2):1249–61. doi: 10.18632/oncotarget.2859 (PMC4359230; doi:10.18632/oncotarget.2859)
Supplement: Supplementary file 1 [file oncotarget-06-1249-s001.pdf]

Histone demethylase RBP2 decreases miR-21 in blast crisis of chronic myeloid leukemia

Supplementary Material

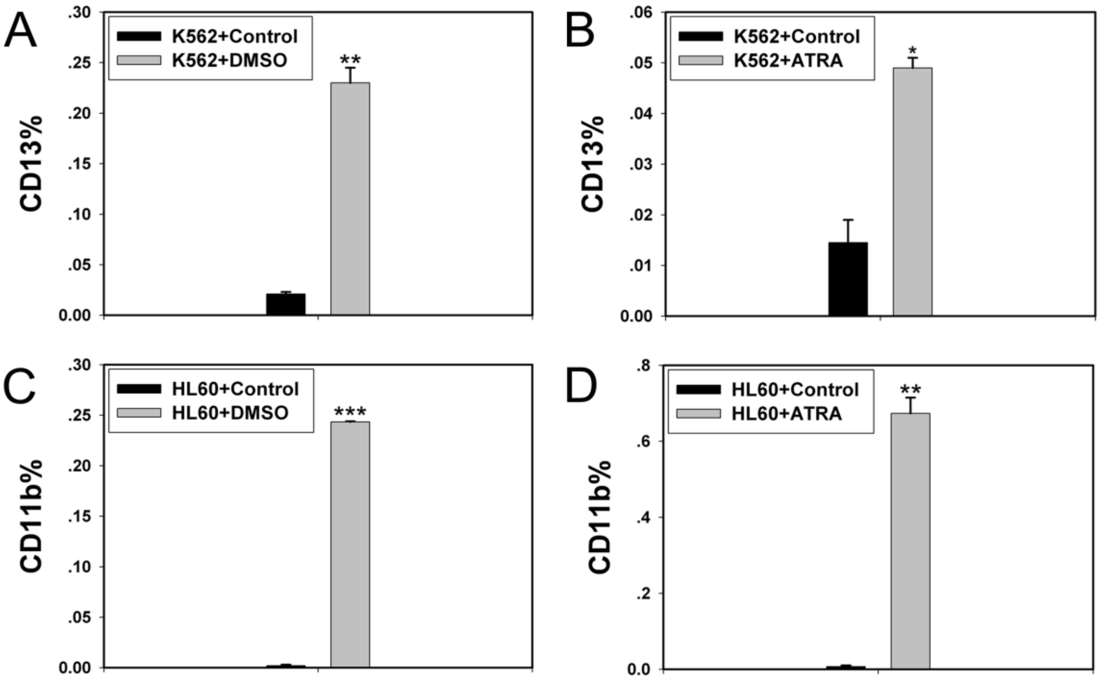

Supplement Figure 1: K562 and HL60 cells were induced to undergo granulocytic differentiation by DMSO or ATRA in vitro. The percentage of CD13 after the treatment with DMSO (A) or ATRA (B) for 7days in K562 cells. Data are mean ± SEM. The percentage of CD11b after the treatment with DMSO (C) or ATRA (D) for 3days in HL60 cells. Data are mean ± SEM. The results were confirmed by 3 independent experiments. *$P < 0.05$, **$P < 0.01$, ***$P < 0.001$.
